# Most amateur football teams do not implement essential components of neuromuscular training to prevent anterior cruciate ligament injuries and lateral ankle sprains

**DOI:** 10.1007/s00167-022-06878-8

**Published:** 2022-02-21

**Authors:** Nikki Rommers, Roland Rössler, Bruno Tassignon, Jo Verschueren, Roel De Ridder, Nicky van Melick, Lieselot Longé, Tim Hendrikx, Peter Vaes, David Beckwée, Christophe Eechaute

**Affiliations:** 1 Department of Clinical Research, University of Basel and University Hospital Basel, Basel, Switzerland; 2 Faculty of Physical Education and Physiotherapy, Department of Movement and Sports Sciences, Vrije Universiteit Brussel, Brussels, Belgium; 3 Department of Sport, Exercise, and Health, University of Basel, Basel, Switzerland; 4 Faculty of Physical Education and Physiotherapy, Human Physiology and Sports Physiotherapy Research Group, Vrije Universiteit Brussel, Brussels, Belgium; 5 Department of Rehabilitation Sciences, Ghent University, Ghent, Belgium; 6 Sports & Orthopedics Research Centre, St. Anna Hospital, Geldrop, The Netherlands; 7 Faculty of Physical Education and Physiotherapy, Rehabilitation Research Group, Vrije Universiteit Brussel, Brussels, Belgium; 8 Department of Rehabilitation Sciences and Physiotherapy, University of Antwerp, Wilrijk, Belgium

**Keywords:** Soccer, ACL, Ankle injury, Neuromuscular training, Barriers

## Abstract

**Purpose:**

Neuromuscular training (NMT) is effective at reducing football injuries. The purpose of this study was to document the use of NMT to prevent anterior cruciate ligament injuries and lateral ankle sprains in adult amateur football and to identify barriers for using NMT.

**Methods:**

A preseason and in-season online survey was completed by players and coaches of 164 football teams. The survey contained questions concerning injury history, type and frequency of NMT, and barriers when NMT was not used.

**Results:**

A total of 2013 players (40% female) and 180 coaches (10% female) completed the preseason survey, whereas 1253 players and 140 coaches completed the in-season survey. Thirty-four percent (preseason) to 21% (in-season) of players used NMT, but only 8% (preseason) to 5% (in-season) performed adequate NMT (i.e. both balance and plyometric exercises, at least twice per week). In the subpopulation of players with an injury history, 12% (preseason) and 7% (in-season) performed adequate NMT. With respect to the coaches, only 5% (preseason) and 2% (in-season) implemented adequate NMT. Most important barriers for using NMT for both players and coaches were a lack of belief in its effectiveness, a lack of knowledge, the belief that stretching is sufficient, and not feeling the need for it.

**Conclusion:**

Most amateur football teams do not implement essential components of NMT. The results highlight the urgent need for developing strategies to enhance the adequate use of NMT in amateur football.

**Level of evidence:**

II.

**Supplementary Information:**

The online version contains supplementary material available at 10.1007/s00167-022-06878-8.

## Introduction

Football is a high-risk sport for lower extremity injuries in all levels of adult play [6]. Anterior cruciate ligament (ACL) injuries and lateral ankle sprains are among the most important injuries in amateur football. These injuries are known for high recurrence rates [14, 31] and negative long-term consequences [10] such as osteoarthritis of the knee [19] and chronic ankle instability [8, 10]. ACL injuries and lateral ankle sprains are also associated with a high medical and socio-economic burden [24]. This substantial personal and societal burden highlights the need for efficacious preventive measures.

Neuromuscular training (NMT) including stabilisation (i.e. jump-landing) and strengthening exercises is effective in reducing the risk for an ACL injury [18, 30] and (recurrent) lateral ankle sprain [9, 29]. The effectiveness of NMT programmes is dependent on the compliance [1, 25]. NMT programmes are of preventive benefit when performed at least twice per week throughout the entire season [18, 29].

Although there is a vast body of literature on the prevention of ACL injuries and lateral ankle sprains, the degree of use has mainly been investigated in controlled study scenarios such as the control arm of an RCT, [1] but scarecely evaluated in a real-life, uncontrolled scenario [7]. Barriers towards the use of prevention programmes have been studied in multiple populations, ranging from professional teams to participants in effectiveness studies regarding injury prevention [4, 16, 17]. It is, however, unknown what the specific barriers are for amateur coaches and players to use preventive NMT. A good understanding of these barriers from the end-user perspective is important to facilitate the use of injury prevention in amateur sport [4, 17].

Therefore, the purpose of this study was to gain more insight into the use of NMT to prevent ACL injuries and lateral ankle sprains in Belgian amateur football in an uncontrolled, real-life scenario. The primary aim was to document detailed aspects of NMT used by amateur football teams to prevent ACL injuries and lateral ankle sprains during the preseason and the in-season and to compare the use of NMT between players with and without a previous injury. The second aim was to identify barriers for using NMT. The hypothesis was that the use of NMT in amateur football teams would be generally low but higher in the subgroup of players with a previous injury. The results of this study can inform medical professionals as well as policy makers about the degree of real-life implementation of effective injury prevention programs in amateur football. Identified barriers can serve as targets to be addressed to increase the use of NMT.

## Materials and methods

This study was performed in accordance with the principles of the Declaration of Helsinki and was approved by the Medical Ethical Committee of the University Hospital Brussels.

During two consecutive seasons (August 2014 and August 2015), a random sample of Belgian amateur football teams (female and male), were contacted via telephone and email. During a personal visit, research assistants informed coaches and players of interested teams about the purpose of the study. To be included, teams had to play in adult regional amateur competition. After providing written informed consent, players and coaches of the included teams were contacted twice (in September and December) to complete an online questionnaire regarding the use of NMT.

### Online survey

In September, players and coaches were invited to complete an online survey (see Appendix 1) regarding the preseason (August–September). This online survey was developed together with stakeholders, such as researchers, coaches, players, physiotherapists, sport physicians, and the national football federation. The survey was pilot tested (informal user acceptance testing) in the target population before the start of the study. All participating players and coaches individually and independently completed the online survey containing demographic information as well as specific information regarding NMT for injury prevention. Players and coaches were independently asked whether or not they used specific exercises during planned training sessions and elsewhere (e.g. at home, or during physiotherapy sessions). If players and coaches used preventive exercises, they were subsequently asked whether the programme contained stabilisation exercises and plyometric exercises and if so, how frequently these exercises were performed, and what the duration of the exercises was. Photos and videos of examples of exercises were shown in the survey to clarify these types of exercises [9, 18, 23, 29, 30]. Players and coaches who did not use NMT were asked about barriers for the use of it. To this end, coaches and players could tick predefined barriers (closed response options), that the research team identified from previous studies [15, 22], and add other barriers (open response option). Four weekly email reminders were sent to the players and coaches who had not yet filled in the questionnaire. Furthermore, team coaches were contacted by the researchers by telephone and were asked to remind their players to complete the questionnaire. To evaluate the test–retest reliability of the questionnaire, a randomly chosen sample of participants completed the questionnaire a second time after 2–3 weeks.

In December of the same season, players and coaches were invited to complete the same online questionnaire again, this to provide information on the use of preventive measures during the first half of the competitive season (in-season phase).

### Statistical analysis

The sample size estimation to observe a difference in the use of injury prevention between players with and without a history of an ACL injury or lateral ankle sprain, was based on an assumed higher use of NMT by 3% in the subgroup of players with a previous injury. Previous literature reported the use of NMT being around 15% [12]. The sample size was determined using a resampling method to be able to show a difference between the two groups using a chi-squared test. Based on simulations, a total sample of 1997 players would be sufficient to detect a difference with a power of 80% and a two-sided type I error rate of 5%.

To evaluate the test–retest reliability of the items of the questionnaire, Cohen’s Kappa was calculated. Concerning the use of NMT, three categories were used: (1) adequate NMT (performing both stabilisation exercises and plyometric exercises at least twice a week in or out of training), (2) inadequate NMT (less than twice a week and/or only one type of exercise) and (3) no NMT. These categories were based on results of systematic reviews and meta-analyses [18, 29]. To assess the association between demographic data such as sex and injury history and the use of NMT, we used a Chi-squared test. The significance level was set at *p* < 0.05. All analyses were performed in R (version 4.0.5).

## Results

Out of 164 recruited teams, a total of 2013 amateur football players (58% response rate) and 180 coaches (72% response rate) completed the questionnaire during the preseason (Table 1). In December of the same season, 62% of these players and 78% of the coaches completed the questionnaire a second time. Appendix 2 presents the flow of participants in the study. Fifty-seven percent of the players reported a history of an ACL injury and/or a lateral ankle sprain in the last two seasons. The test–retest reliability of the responses (*n* = 58 participants) was acceptable to excellent with Kappa coefficients ranging from 0.55 to 1.00.

**Table 1:** Demographic characteristics of the players and coaches

	Preseason	In-season
**Players**	***N* = 2013**	***N* = 1253**
Age (years)*	24.0 (5.5)	24.2 (5.3)
Training frequency (per week)*	2.1 (0.5)	2.1 (0.5)
Female players†	808 (40.1%)	487 (38.9%)
History of lateral ankle sprain†	1018 (50.6%)	589 (47%)
History of ACL injury†	237 (11.9%)	156 (12.5%)
Involved in other sport†	889 (44.2%)	559 (44.6%)
Use of tape/brace during training/match†	309 (15.4%)	199 (15.9%)
**Coaches**	***N* = 180**	***N* = 140**
Age (years)*	43.1 (10.0)	44.0 (10.7)
Years of experience*	14.1 (9.4)	15.6 (10.1)
Training frequency (per week)*	2.0 (0.5)	2.0 (0.5)
Female coaches†	18 (10.0%)	14 (10.0%)
Training education certificate†	88 (48.9%)	67 (47.9%)
History of lateral ankle sprain†	116 (64.4%)	89 (63.5%)
History of ACL injury†	35 (19.4%)	27 (19.3%)

*ACL* anterior cruciate ligament.

*Mean (standard deviation), †: *n* (%)

### Players

**Fig. 1:**
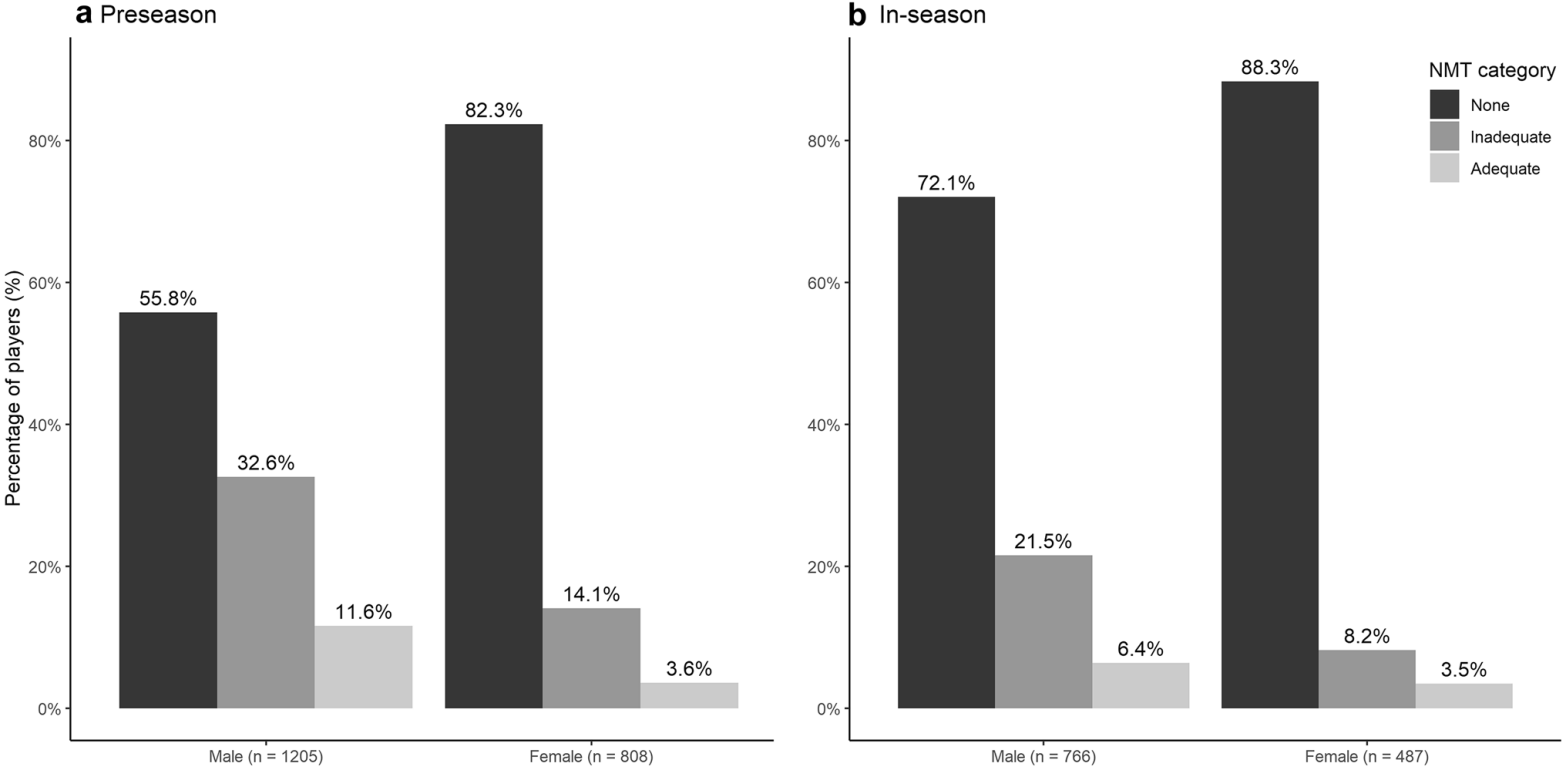
Use of NMT in male and female football players during preseason (**a**) and in-season (**b**)

Thirty-eight percent of all players (*n* = 762) used NMT for injury prevention during the preseason. In-season, this percentage was 28% (*n* = 353). Of all players, 8% (preseason) and 5% (in-season) used adequate NMT. The number of male players using adequate NMT was significantly higher compared to female players (preseason: *X*-squared 47.2, *p* < 0.001; in-season: *X*-squared 47.103, *p* < 0.001) (Fig. 1). A detailed overview of the responses to the survey questions during the preseason and in-season is presented in Table 2. Out of the 38% of players who did some kind of prevention, 73.9% performed stabilisation exercises and 63.6% performed plyometric exercises during the preseason, but mostly not combined, less than twice a week and less than 10 min per session. In-season, these numbers were lower.

**Table 2:** Detailed results of NMT use by players, stratified by sex and injury history

		**Preseason**					**In-season**				
		Overall	Male	Female	No injury history	Injury history	Overall	Male	Female	No injury history	Injury history
N/n		2013	1205	808	873	1140	1253	766	487	587	666
Any injury prevention (%)	Yes	762 (37.9)	576 (47.8)	186 (23.0)	240 (27.5)	522 (45.8)	353 (28.2)	266 (34.7)	87 (17.9)	126 (21.5)	227 (34.1)
	No	1251 (62.1)	629 (52.2)	622 (77.0)	633 (72.5)	618 (54.2)	900 (71.8)	500 (65.3)	400 (82.1)	461 (78.5)	439 (65.9)
Stabilisation exercises (%)	Yes	563 (73.9)	446 (77.4)	117 (62.9)	161 (67.1)	402 (77.0)	225 (63.7)	168 (63.2)	57 (65.5)	72 (57.1)	153 (67.4)
	No	199 (26.1)	130 (22.6)	69 (37.1)	79 (32.9)	120 (23.0)	128 (36.3)	98 (36.8)	30 (34.5)	54 (42.9)	74 (32.6)
Attention to correct landing technique during stabilisation exercises (%)	Yes	435 (78.1)	342 (77.7)	93 (79.5)	125 (78.6)	310 (77.9)	186 (82.7)	142 (84.5)	44 (77.2)	63 (87.5)	123 (80.4)
	No	122 (21.9)	98 (22.3)	24 (20.5)	34 (21.4)	88 (22.1)	39 (17.3)	24 (15.5)	13 (22.8)	9 (12.5)	30 (19.6)
Stabilisation exercises during training (%)	Yes	347 (61.6)	284 (63.7)	63 (53.8)	111 (68.9)	236 (58.7)	115 (51.1)	95 (56.5)	20 (35.1)	45 (62.5)	70 (45.8)
	No	216 (38.4)	162 (36.3)	54 (46.2)	50 (31.1)	166 (41.3)	110 (48.9)	73 (43.5)	37 (64.9)	27 (37.5)	83 (54.2)
Frequency of stabilisation exercises during training (%)	< 1 time/week	42 (12.2)	30 (10.6)	12 (19.0)	16 (14.7)	26 (11.0)	29 (25.2)	21 (22.1)	8 (40.0)	15 (33.3)	14 (20.0)
	1 time/week	165 (47.8)	136 (48.2)	29 (46.0)	58 (53.2)	107 (45.3)	52 (45.2)	46 (48.4)	6 (30.0)	19 (42.2)	33 (47.1)
	2 times/week	121 (35.1)	102 (36.2)	19 (30.2)	30 (27.5)	91 (38.6)	29 (25.2)	24 (25.3)	5 (25.0)	9 (20.0)	20 (28.6)
	3 times/week	14 ( 4.1)	12 ( 4.3)	2 ( 3.2)	4 ( 3.7)	10 ( 4.2)	5 ( 4.3)	4 ( 4.2)	1 ( 5.0)	2 ( 4.4)	3 ( 4.3)
	4 times/week	2 ( 0.6)	2 ( 0.7)	0 ( 0.0)	1 ( 0.9)	1 ( 0.4)	0 ( 0.0)	0 ( 0.0)	0 ( 0.0)	0 ( 0.0)	0 ( 0.0)
	5 times/week	1 ( 0.3)	0 ( 0.0)	1 ( 1.6)	0 ( 0.0)	1 ( 0.4)	0 ( 0.0)	0 ( 0.0)	0 ( 0.0)	0 ( 0.0)	0 ( 0.0)
Duration of stabilisation exercises during training (%)	< 10 min	242 (69.9)	193 (68.2)	49 (77.8)	83 (74.8)	159 (67.7)	87 (75.7)	71 (74.7)	16 (80.0)	35 (77.8)	52 (74.3)
	> = 10 min	104 (30.1)	90 (31.8)	14 (22.2)	28 (25.2)	76 (32.3)	28 (24.3)	24 (25.3)	4 (20.0)	10 (22.2)	18 (25.7)
Stabilisation exercises at home (%)	Yes	356 (63.6)	288 (65.0)	68 (58.1)	79 (49.4)	277 (69.2)	158 (70.2)	113 (67.3)	45 (78.9)	42 (58.3)	116 (75.8)
	No	204 (36.4)	155 (35.0)	49 (41.9)	81 (50.6)	123 (30.8)	67 (29.8)	55 (32.7)	12 (21.1)	30 (41.7)	37 (24.2)
Frequency stabilisation exercises at home (%)	< 1 time/week	16 ( 4.5)	12 ( 4.2)	4 ( 5.9)	8 (10.1)	8 ( 2.9)	11 ( 7.0)	7 ( 6.2)	4 ( 8.9)	4 ( 9.5)	7 ( 6.0)
	1 time/week	105 (29.5)	87 (30.2)	18 (26.5)	24 (30.4)	81 (29.2)	54 (34.2)	42 (37.2)	12 (26.7)	15 (35.7)	39 (33.6)
	2 times/week	140 (39.3)	115 (39.9)	25 (36.8)	31 (39.2)	109 (39.4)	50 (31.6)	36 (31.9)	14 (31.1)	11 (26.2)	39 (33.6)
	3 times/week	60 (16.9)	49 (17.0)	11 (16.2)	11 (13.9)	49 (17.7)	35 (22.2)	24 (21.2)	11 (24.4)	10 (23.8)	25 (21.6)
	4 times/week	9 ( 2.5)	8 ( 2.8)	1 ( 1.5)	1 ( 1.3)	8 ( 2.9)	4 ( 2.5)	2 ( 1.8)	2 ( 4.4)	1 ( 2.4)	3 ( 2.6)
	5 times/week	13 ( 3.7)	9 ( 3.1)	4 ( 5.9)	2 ( 2.5)	11 ( 4.0)	1 ( 0.6)	0 ( 0.0)	1 ( 2.2)	0 ( 0.0)	1 ( 0.9)
	> = 6 times/week	13 ( 3.7)	8 ( 2.8)	5 ( 7.4)	2 ( 2.5)	11 ( 4.0)	3 ( 1.9)	2 ( 1.8)	1 ( 2.2)	1 ( 2.4)	2 ( 1.7)
Duration of stabilisation exercises at home (%)	< 10 min	159 (44.5)	126 (43.6)	33 (48.5)	35 (43.8)	124 (44.8)	78 (49.4)	53 (46.9)	25 (55.6)	16 (38.1)	62 (53.4)
	> = 10 min	198 (55.5)	163 (56.4)	35 (51.5)	45 (56.2)	153 (55.2)	80 (50.6)	60 (53.1)	20 (44.4)	26 (61.9)	54 (46.6)
Plyometric exercises (%)	Yes	484 (63.6)	396 (68.9)	88 (47.3)	143 (59.6)	341 (65.5)	186 (59.8)	161 (60.5)	25 (55.6)	53 (54.1)	133 (62.4)
	No	277 (36.4)	179 (31.1)	98 (52.7)	97 (40.4)	180 (34.5)	125 (40.2)	105 (39.5)	20 (44.4)	45 (45.9)	80 (37.6)
Attention to correct landing technique during plyometric exercises (%)	Yes	290 (60.3)	304 (77.0)	69 (80.2)	108 (76.1)	265 (78.2)	162 (87.1)	137 (85.1)	20 (80)	44 (83.0)	103 (77.4)
	No	191 (39.7)	91 (23.0)	17 (19.8)	34 (23.9)	74 (21.8)	24 (12.9)	24 (14.9)	5 (20)	9 (17.0)	30 (22.6)
Plyometric exercises during training (%)	Yes	290 (60.3)	247 (62.8)	43 (48.9)	86 (61.0)	204 (60.0)	117 (56.8)	92 (57.1)	25 (55.6)	34 (58.6)	83 (56.1)
	No	191 (39.7)	146 (37.2)	45 (51.1)	55 (39.0)	136 (40.0)	89 (43.2)	69 (42.9)	20 (44.4)	24 (41.4)	65 (43.9)
Frequency of plyometric exercises during training (%)	< 1 time/week	47 (16.0)	40 (15.9)	7 (16.3)	13 (14.9)	34 (16.4)	31 (22.6)	31 (33.7)	0 ( 0.0)	11 (28.2)	20 (20.4)
	1 time/week	172 (58.5)	144 (57.4)	28 (65.1)	56 (64.4)	116 (56.0)	64 (46.7)	39 (42.4)	25 (55.6)	16 (41.0)	48 (49.0)
	2 times/week	64 (21.8)	58 (23.1)	6 (14.0)	15 (17.2)	49 (23.7)	39 (28.5)	19 (20.7)	20 (44.4)	12 (30.8)	27 (27.6)
	3 times/week	9 ( 3.1)	8 ( 3.2)	1 ( 2.3)	3 ( 3.4)	6 ( 2.9)	3 ( 2.2)	3 ( 3.3)	0 ( 0.0)	0 ( 0.0)	3 ( 3.1)
	4 times/week	1 ( 0.3)	1 ( 0.4)	0 ( 0.0)	0 ( 0.0)	1 ( 0.5)	0 ( 0.0)	0 ( 0.0)	0 ( 0.0)	0 ( 0.0)	0 ( 0.0)
	5 times/week	1 ( 0.3)	0 ( 0.0)	1 ( 2.3)	0 ( 0.0)	1 ( 0.5)	0 ( 0.0)	0 ( 0.0)	0 ( 0.0)	0 ( 0.0)	0 ( 0.0)
Duration of plyometric exercises during training (%)	< 10 min	192 (65.5)	161 (64.4)	31 (72.1)	61 (69.3)	131 (63.9)	84 (75.0)	69 (75.0)	15 (75.0)	31 (81.6)	53 (71.6)
	> = 10 min	101 (34.5)	89 (35.6)	12 (27.9)	27 (30.7)	74 (36.1)	28 (25.0)	23 (25.0)	5 (25.0)	7 (18.4)	21 (28.4)
Plyometric exercises at home (%)	Yes	215 (44.4)	174 (43.9)	41 (46.6)	49 (34.3)	166 (48.7)	111 (54.4)	89 (55.3)	22 (51.2)	31 (45.6)	80 (58.8)
	No	269 (55.6)	222 (56.1)	47 (53.4)	94 (65.7)	175 (51.3)	93 (45.6)	72 (44.7)	21 (48.8)	37 (54.4)	56 (41.2)
Frequency of plyometric exercises at home (%)	< 1 time/week	12 ( 5.6)	10 ( 5.7)	2 ( 4.9)	3 ( 6.1)	9 ( 5.4)	13 (11.7)	10 (11.2)	3 (13.6)	3 ( 9.7)	10 (12.5)
	1 time/week	64 (29.6)	53 (30.3)	11 (26.8)	19 (38.8)	45 (26.9)	50 (45.0)	46 (51.7)	4 (18.2)	14 (45.2)	36 (45.0)
	2 times/week	87 (40.3)	73 (41.7)	14 (34.1)	15 (30.6)	72 (43.1)	32 (28.8)	25 (28.1)	7 (31.8)	10 (32.3)	22 (27.5)
	3 times/week	40 (18.5)	31 (17.7)	9 (22.0)	8 (16.3)	32 (19.2)	13 (11.7)	7 ( 7.9)	6 (27.3)	3 ( 9.7)	10 (12.5)
	4 times/week	4 ( 1.9)	4 ( 2.3)	0 ( 0.0)	0 ( 0.0)	4 ( 2.4)	2 ( 1.8)	1 ( 1.1)	1 ( 4.5)	0 ( 0.0)	2 ( 2.5)
	5 times/week	4 ( 1.9)	2 ( 1.1)	2 ( 4.9)	1 ( 2.0)	3 ( 1.8)	0 ( 0.0)	0 ( 0.0)	0 ( 0.0)	0 ( 0.0)	0 ( 0.0)
	> = 6 times/week	5 ( 2.3)	2 ( 1.1)	3 ( 7.3)	3 ( 6.1)	2 ( 1.2)	1 ( 0.9)	0 ( 0.0)	1 ( 4.5)	1 ( 3.2)	0 ( 0.0)
Duration of plyometric exercises at home (%)	< 10 min	88 (40.7)	67 (38.3)	21 (51.2)	21 (42.9)	67 (40.1)	63 (56.8)	49 (55.1)	14 (63.6)	14 (45.2)	49 (61.3)
	> = 10 min	128 (59.3)	108 (61.7)	20 (48.8)	28 (57.1)	100 (59.9)	48 (43.2)	40 (44.9)	8 (36.4)	17 (54.8)	31 (38.8)
Tape/brace (%)	No tape/brace	1702 (84.6)	1051 (87.2)	651 (80.8)	806 (92.4)	896 (78.7)	1054 (84.1)	660 (86.2)	394 (80.9)	548 (93.4)	506 (76.0)
	Tape/brace	309 (15.4)	154 (12.8)	155 (19.2)	66 ( 7.6)	243 (21.3)	199 (15.9)	106 (13.8)	93 (19.1)	39 ( 6.6)	160 (24.0)

### Players with a previous ACL injury or lateral ankle sprain

**Fig. 2:**
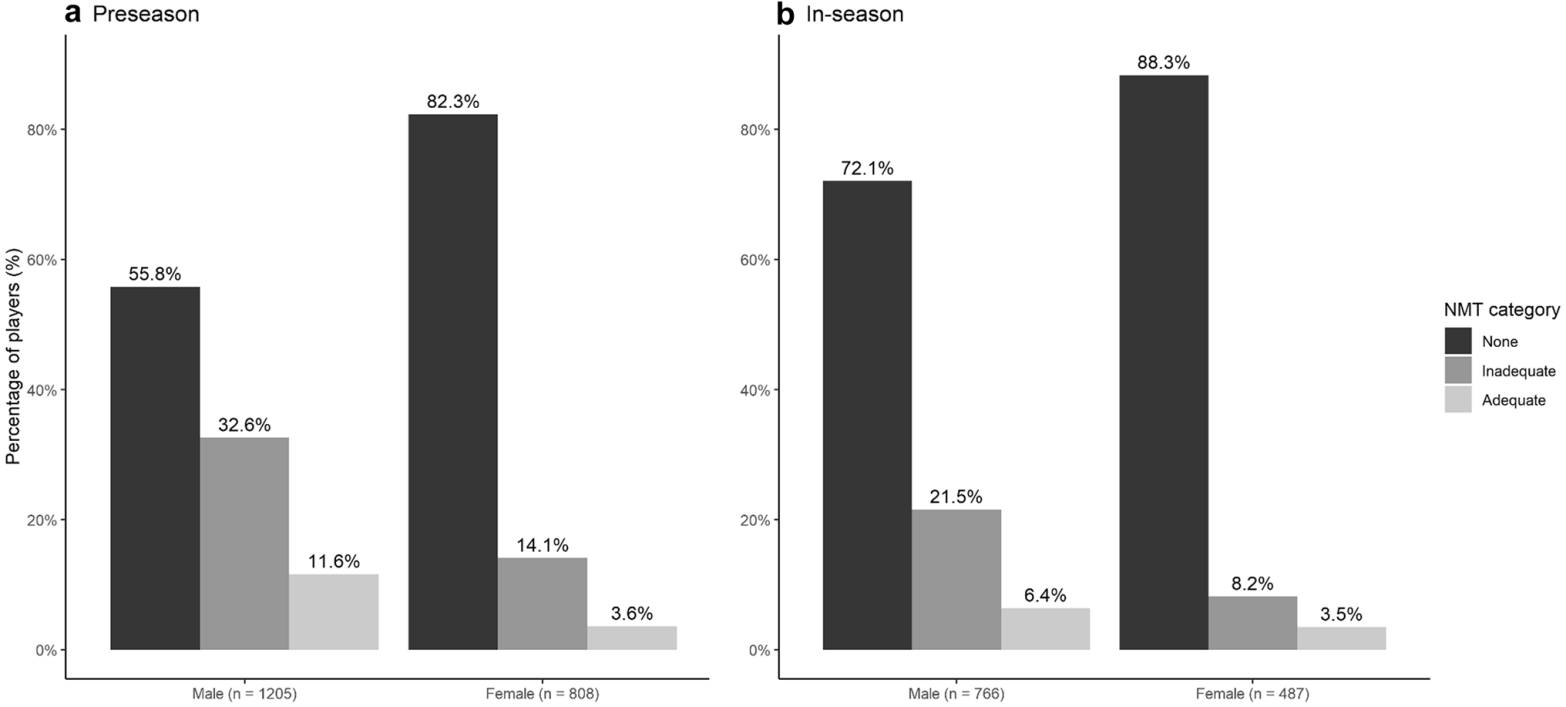
Distribution of players with a previous lateral ankle sprain and/or ACL injury among the prevention categories during preseason (*n* = 1140) and in-season (*n* = 666)

Forty-six percent of players with a previous ACL injury or lateral ankle sprain used NMT during the preseason, compared to 34% in-season. Twelve percent (preseason) and 7% (in-season) used adequate NMT (Fig. 2). Players with a previous injury performed significantly more NMT than players without a previous injury (preseason *X*-squared 36.9, *p* < 0.001, in-season *X*-squared 80.3, *p* < 0.001). Table 2 shows the detailed responses of players stratified by injury history. Similar results were found in the subpopulations with either a history of ACL injury or lateral ankle sprain.

### Coaches

**Fig. 3:**
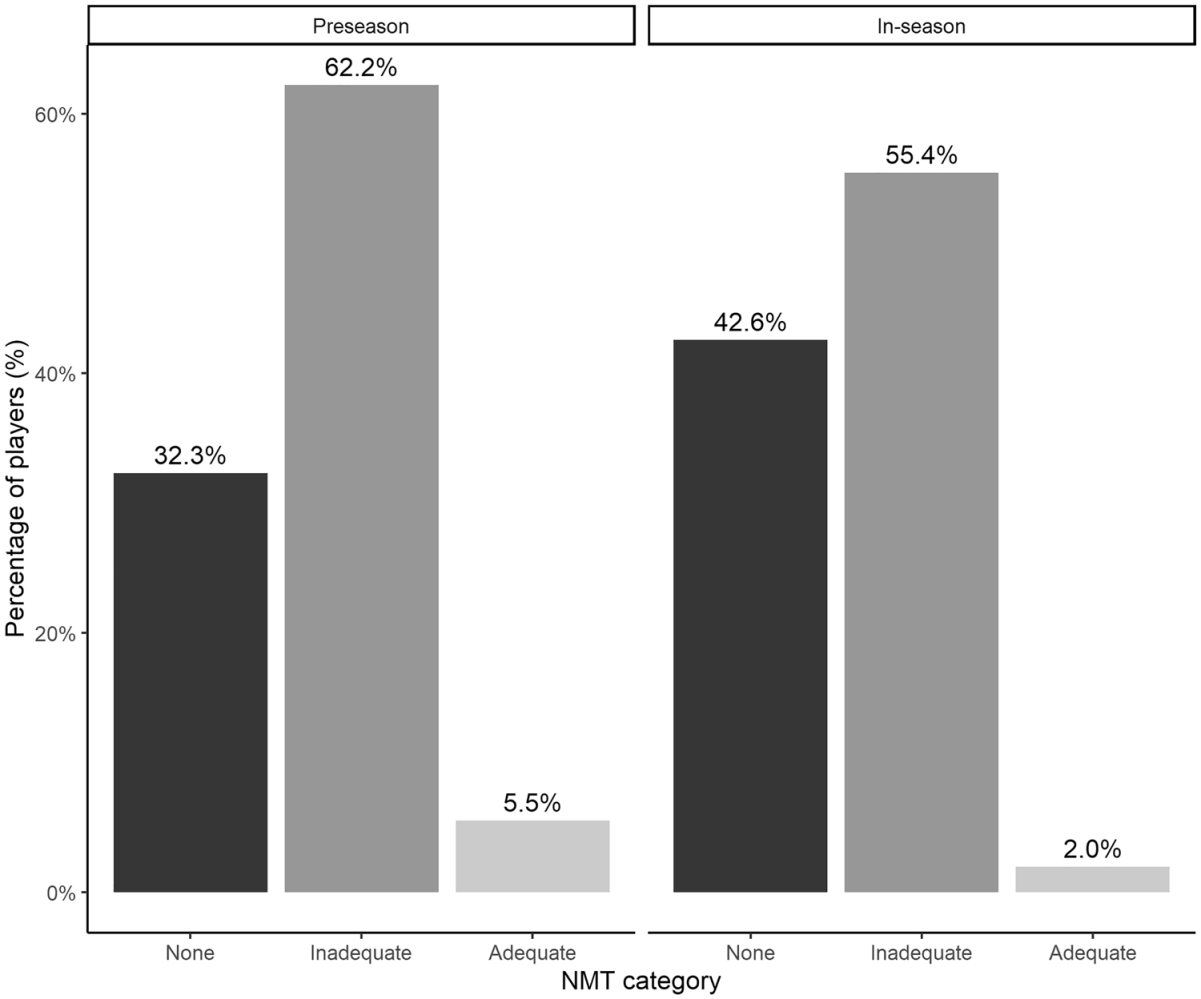
Use of prevention among coaches during the preseason (*n* = 180) and in-season (*n* = 140)

During the preseason, 76% of the coaches implemented NMT compared to 62% in-season. Six percent (preseason) to 2% (in-season) of the coaches used adequate NMT (Fig. 3). Three percent of coaches with a history of an ACL injury or lateral ankle sprain themselves implemented adequate NMT during their training sessions. This number was significantly higher when compared to coaches without a history of injury (1%) (*X*-squared 12.9, *p* = 0.002). A detailed overview of the coaches’ responses to the survey questions during the preseason and in-season is displayed in Table 3.

**Table 3:** Detailed results of NMT implementation by coaches

		Preseason	In-season
*N*		180	140
Prevention (%)	Yes	114 (63.3)	74 (52.9)
	No	66 (36.7)	66 (47.1)
Stababilisation exercises (%)	Yes	78 (68.4)	48 (64.9)
	No	36 (31.6)	26 (35.1)
Attention to correct landing technique during stabilisation exercises (%)	Yes	52 (66.7)	37 (77.1)
	No	26 (33.3)	11 (22.9)
Frequency of stabilisation exercises (%)	< 1 time/week	13 (16.7)	30 (62.5)
	1 time/week	41 (52.6)	12 (25.0)
	2 times/week	20 (25.6)	6 (12.5)
	3 times/week	2 ( 2.6)	0 ( 0.0)
	4 times/week	2 ( 2.6)	0 ( 0.0)
Duration of stabilisation exercises (%)	< 10 min	54 (69.2)	21 (43.8)
	> = 10 min	24 (30.8)	27 (56.2)
Plyometric exercises (%)	Yes	79 (69.3)	56 (75.7)
	No	35 (30.7)	18 (24.3)
Attention to correct landing technique during plyometric exercises (%)	Yes	54 (67.5)	42 (75.0)
	No	26 (32.5)	14 (25.0)
Frequency of plyometric exercises (%)	< 1 time/week	19 (24.1)	33 (58.9)
	1 time/week	47 (59.5)	20 (35.7)
	2 times/week	11 (13.9)	3 ( 5.4)
	3 times/week	1 ( 1.3)	0 ( 0.0)
	4 times/week	1 ( 1.3)	0 ( 0.0)
Duration of plyometric exercises (%)	< 10 min	57 (72.2)	32 (57.1)
	> = 10 min	22 (27.8)	24 (42.9)

### Barriers for the use of NMT

**Fig. 4:**
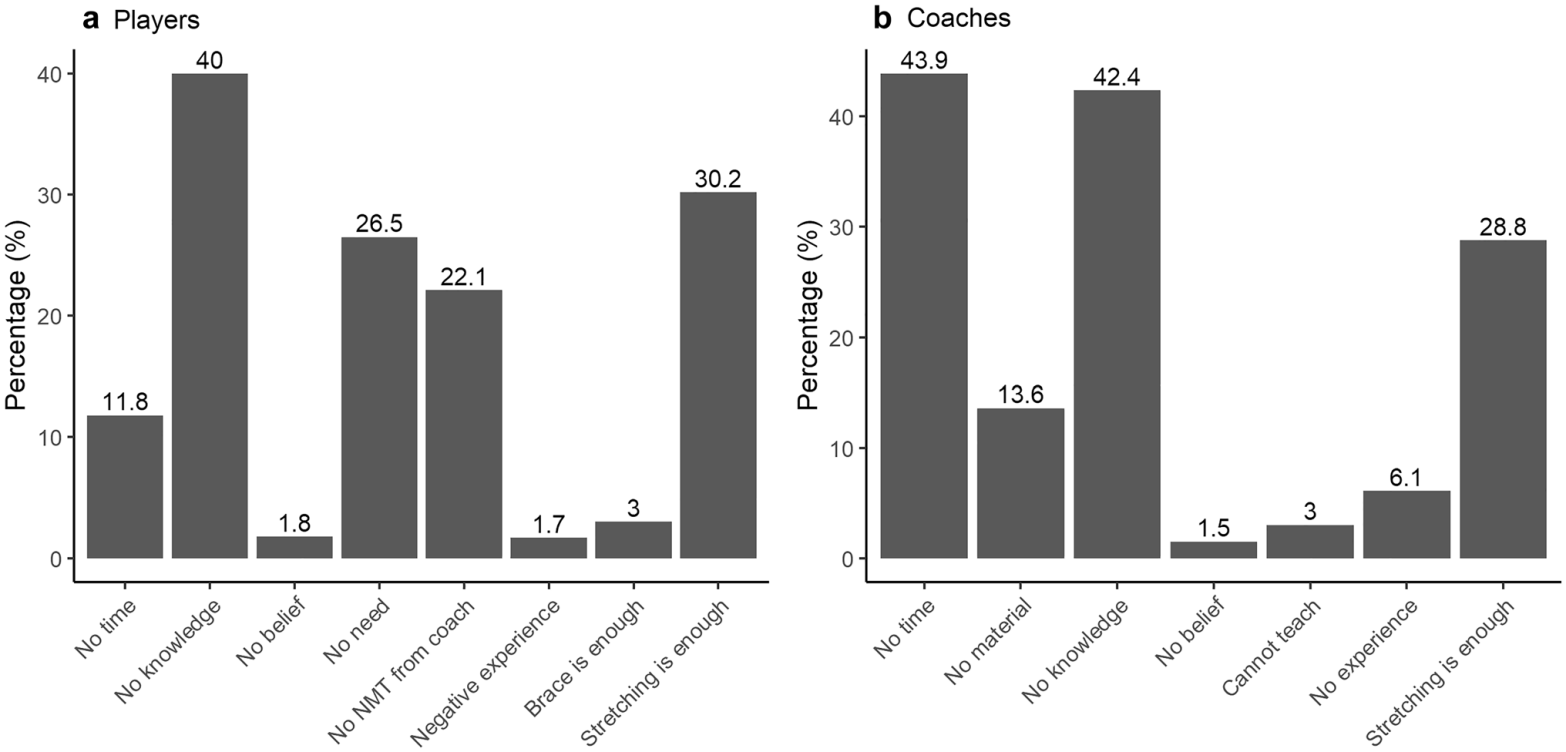
Players’ (**a**) and coaches’ (**b**) barriers for the use of NMT

The barriers for using NMT by players and coaches are presented in Fig. 4. The most common barrier for players was a lack of knowledge and for coaches a lack of time.

## Discussion

The most important finding of the present study was that only a very small proportion of amateur football players and coaches used NMT in an adequate way. The same results were observed in the subpopulation of players with a history of an ACL or lateral ankle sprain. The most important barriers for using NMT were a lack of knowledge (players and coaches), the belief that stretching was sufficient (players and coaches), not feeling the need for NMT (players), and a lack of time (coaches).

### NMT

In this study, almost 60% of the players already sustained an ACL injury and/or a lateral ankle sprain in the past two seasons. Despite the evidence for the effectiveness of preventive exercises to reduce the risk of recurrency of these injuries [9, 18], only 12% of the players in the present cohort performed adequate NMT. It could be assumed that the players with a previous lateral ankle sprain who did not use NMT would take other effective preventive measures, such as using an external support (e.g. brace) [2]. However, in the subgroup of players with a previous lateral ankle sprain, only 16% used a tape or brace and 49% did not use any kind of preventive measure (see Table 2).

Similar to the data of the players, only a very small proportion of the coaches in this study implemented adequate NMT during their training. Gebert et al. (2019) [7] reported that only 20% of the amateur football coaches use a prevention programme at least once per week. This is in accordance with the preseason findings. In-season, more than half of the coaches incorporated stabilisation or plyometric exercises during their training sessions less than once per week (Table 3). The observation that only half of the coaches in this study disposed a formal trainer education certificate may at least partially explain the low number of coaches adequately using NMT. A previous study in amateur youth football found that the lower level of coach education was associated with a more negative attitude towards using injury prevention programmes [3].

The criteria for adequate NMT were based on the results of meta-analyses regarding the effectiveness of exercise-based injury prevention [9, 11, 18]. Furthermore, previous studies concluded that NMT should be performed both during preseason and in-season to induce the desired prophylactic effect [9, 11, 18].

It has been demonstrated that the effectiveness of injury prevention programmes depends on the degree of compliance [28] and that the effectiveness increases with increasing compliance to such programmes [21, 32]. Studies investigating the effect of “11 + Kids” and “11 + ” programmes in amateur football players, mention that these programmes need to be performed at least twice per week throughout the whole season [21, 25]. However, the already very low proportion of players and coaches using adequate NMT (or any NMT) during the preseason dropped during the competitive season. This general trend over the course of the season is present for both stabilisation and plyometric exercises, and for the frequency of execution as well as duration of exercises during training (Table 2). Future studies should therefore focus on the continuation of NMT throughout the entire football season and the reasons for discontinuation once the competition starts.

### Barriers for NMT

One of the most important barriers for using NMT is the lack of knowledge of how NMT should be incorporated adequately during training (e.g. essential components and frequency). The relatively high proportion of coaches who reported not to know which exercises to use or simply stated lacking time is in accordance with previous studies [20, 27]. Furthermore, a vast amount of coaches and players believed that stretching is sufficient to prevent ACL injuries and lateral ankle sprains, although this is not supported by literature [13, 18]. This finding suggests that much more efforts are needed to educate coaches and players regarding injury prevention. Another important barrier for players to use NMT was the perception that they do not need preventive exercises. This is remarkable since a large proportion of the players and coaches participating in this study reported already having sustained a previous ACL injury or lateral ankle sprain in the last two seasons. As one may suppose that a large proportion of these players visited medical doctors and underwent rehabilitation, one would expect that they should be more aware of a potential injury risk and the benefits of NMT. Informing players about NMT is a task that should be taken up by clinicians to improve the degree of compliance and reduce recurrent injuries.

### Practical application and future direction

The awareness and knowledge of the benefits and content of NMT needs to be increased in both players and coaches, as supported by the lack of adequate use of NMT in amateur football teams. A previous coach survey in several European countries showed that the application of injury prevention in the different countries was associated with the proportion of coaches that received formal training [3]. It could, therefore, be of interest to provide (free) workshops for coaches as well as players to enhance knowledge and work together with all stakeholders in the design of feasible and effective injury prevention measures [26]. In formal coach education, there should be more emphasis on injury prevention. The presence of barriers such as lack of knowledge and lack of belief in effectiveness indicate the important role of federations and clinicians in injury prevention. National and international federations should cooperate with amateur football representatives to set up education initiatives, and co-create feasible injury prevention initiatives. The existing RE-AIM framework could help to target the issue of implementation in amateur sport settings [5, 26]. The findings of the present study should encourage athletic trainers and clinicians to determine the perception of amateur football players concerning the use of preventive measures to counter possible barriers for NMT and to increase its use.

This study is the first to document in a detailed way the use of NMT and associated barriers perceived by coaches and players in Belgian amateur football. The large and representative sample provides a comprehensive picture of the use of NMT in this population. However, this study has some limitations. The first limitation is the self-reported nature of the data. Since the use of NMT was investigated during preseason and in-season, respectively, there are no data on other periods of the season. However, as results of the preseason are very similar to those of the first half of the competitive season, it seems unlikely that the use of NMT during the second half of the competitive season would significantly change. Furthermore, a potential recall bias could not be ruled out. Due to the sample size and geographic spreading, the data could not be verified “on the field”. Therefore, the possibility of participants giving socially desirable answers could not be ruled out. However, Kappa coefficients of the items of the online survey demonstrate acceptable to excellent test–retest reliability of the questionnaire. As expected and as it is usual in survey studies, a substantial proportion of the initial sample did not fill in the survey a second time in December. However, there were no significant differences between the participants at two time points, suggesting that the drop out was not selective.

## Conclusion

Most amateur football teams do not implement essential components of NMT to prevent ACL injuries and lateral ankle sprains, neither during the preseason nor in-season. More importantly, the majority of the players with a previous ACL injury or lateral ankle sprain and thus being at increased risk, neither used adequate NMT, used a brace or tape. Most important barriers for using NMT were a lack of knowledge, the belief that stretching is sufficient, and not feeling the need for using NMT.

## Supplementary Information

Below is the link to the electronic supplementary material.

Supplementary file1 (DOCX 1449 KB)

## Data Availability

The data are available upon reasonable request to the corresponding author.

## Code avialability

Data were mainly analyzed descriptively in R using packages dplyr and ggplot2. The codes for data processing and descriptive statistics as well as plots are available upon request to the corresponding author.
