# Supplementary material for: Most amateur football teams do not implement essential components of neuromuscular training to prevent anterior cruciate ligament injuries and lateral ankle sprains
Source: Knee Surg Sports Traumatol Arthrosc. 2022 Feb 21;30(4):1169–79. doi: 10.1007/s00167-022-06878-8 (PMC9007793; doi:10.1007/s00167-022-06878-8)

**Appendix 1**. English translation of the questionnaires used in this study (original questionnaires were in Dutch).

**Questionnaire players**

1. Age
2. Sex
   - Male
   - Female
3. How many years do you play football?
4. What is your highest level of play?
   - BeNeliga/Pro league
   - 1^st^ national amateur league
   - 2^nd^ national amateur league
   - 3^rd^ national amateur league
   - 4^th^ national amateur league
   - 1^st^ provincial league
   - 2^nd^ provincial league
   - 3^rd^ provincial league
   - 4^th^ provincial league
5. How many times per week do you have football training?
   - 1 x/week
   - 2 x/week
   - 3 x/week
   - > 3 x/week
6. Do you also practice other organised sports?
   - Yes
   - No
7. Did you ever suffer from a lateral ankle sprain because of which you were not able to practice your sport for a certain time, because of which you consulted a medical doctor, or because of which you had physiotherapy treatment?
   - Yes, once
   - Yes, multiple times
   - No
8. When was your last ankle sprain?
   - < 1 month ago
   - 1-3 months ago
   - 3-6 months ago
   - 6-12 months ago
   - 1-2 years ago
   - > 2 years ago
9. Did you ever suffer an anterior cruciate ligament tear?
   - Yes, once
   - Yes, multiple times
   - No
10. When was your last anterior cruciate ligament tear?
    - < 1 month ago
    - 1-3 months ago
    - 3-6 months ago
    - 6-12 months ago
    - 1-2 years ago
    - > 2 years ago

Injury prevention

1. Did you perform exercises to prevent ankle or knee injuries during the preseason (July-August) / in-season (September-November)?
   - Yes
   - No (go to question 29)
2. Did you perform exercises to improve your stability? (see video for an example exercise)
   - Yes
   - No (go to question 20)
3. Did you use special material (see photo right) during
   the execution of the stability exercises?
   - Yes
   - No
4. Did you perform the stability exercises during training?
   - Yes
   - No (go to question 17)
5. How often did you perform stability exercises?
   - 1 x/week
   - 2 x/week
   - 3 x/week
   - 4 x/week
   - 5 x/week
   - 1 x/month
   - 2 x/month
   - 3 x/month
6. How much time did you spend on stability exercises during training each time?
   - < 10 minutes
   - > 10 minutes
7. Did you perform stability exercises outside of training (at home, in the gym, at the physiotherapist,…)?
   - Yes
   - No (go to question 20)
8. How often did you perform stability exercises outside of training?
   - 1 x/week
   - 2 x/week
   - 3 x/week
   - 4 x/week
   - 5 x/week
   - 1 x/month
   - 2 x/month
   - 3 x/month
9. How much time did you spend each time on stability exercises outside of training?
   - < 10 minutes
   - > 10 minutes
10. Did you perform exercises to improve your leg strength by means of jumping exercises, also called plyometric exercises? (see video for an example exercise)
    - Yes
    - No (go to question 27)
11. Did you perform the plyometric exercises during training?
    - Yes
    - No (go to question 24)
12. How often did you perform plyometric exercises?
    - 1 x/week
    - 2 x/week
    - 3 x/week
    - 4 x/week
    - 5 x/week
    - 1 x/month
    - 2 x/month
    - 3 x/month
13. How much time did you spend on plyometric exercises during training each time?
    - < 10 minutes
    - > 10 minutes
14. Did you perform plyometric exercises outside of training (at home, in the gym, at the physiotherapist,…)?
    - Yes
    - No (go to question 27)
15. How often did you perform plyometric exercises outside of training?
    - 1 x/week
    - 2 x/week
    - 3 x/week
    - 4 x/week
    - 5 x/week
    - 1 x/month
    - 2 x/month
    - 3 x/month
16. How much time did you spend each time on plyometric exercises outside of training?
    - < 10 minutes
    - > 10 minutes
17. If you performed other exercises to prevent ankle and/or knee injuries than described in the questions above, please describe the exercises you performed.
18. Where did you get knowledge about exercises to prevent ankle or knee injuries?
    - Coach
    - Physiotherapist
    - Medical doctor
    - Internet
    - (scientific) journals
    - Info session of the football federation
    - Info brochure of the football federation
    - Other (specify
    - None of the above
19. Please tick all reasons that apply to you for not performing exercises to prevent ankle or knee injuries.
    - I do not need preventive exercises
    - I do not believe preventive exercises could prevent injuries
    - I have no knowledge of preventive exercises
    - I do not have positive experiences from performing preventive exercises in the past
    - My doctor or physiotherapist told me these exercises cannot prevent injuries
    - My coach told me these exercises cannot prevent injuries
    - My coach did not implement preventive exercises
    - I have no time for preventive exercises
    - I believe that a tape or brace is sufficient to prevent injuries
    - I think that stretching and warming-up are sufficient to prevent injuries
    - Other (specify)
20. Do you use a tape or brace to protect your knee and/or ankle
    - Yes, for my ankle
    - Yes, for my knee
    - Yes, for both ankle and knee
    - No
21. When do you use a tape or brace?
    - Only during training
    - Only during matches
    - During both training and matches

**Questionnaire coaches**

1. Age
2. Sex
   - Male
   - Female
3. How many years are you coaching a football team?
4. What coaching certificate do you have?
   - None
   - Initiator
   - Instructor
   - Trainer B (UEFA B)
   - Trainer A (elite youth)
   - Trainer A (senior)
   - Other (specify)
5. Did you play football yourself?
   - Yes
   - No
6. What is your highest level of play?
   - BeNeliga/Pro league
   - 1^st^ national amateur league
   - 2^nd^ national amateur league
   - 3^rd^ national amateur league
   - 4^th^ national amateur league
   - 1^st^ provincial league
   - 2^nd^ provincial league
   - 3^rd^ provincial league
   - 4^th^ provincial league
7. How many times per week do you train your football team?
   - 1 x/week
   - 2 x/week
   - 3 x/week
   - > 3 x/week
8. What is the duration of a training session?
   - 1 hour
   - 1-2 hours
   - > 2 hours
9. Did you ever suffer from a lateral ankle sprain because of which you were not able to practice your sport for a certain time, because of which you consulted a medical doctor, or because of which you had physiotherapy treatment?
   - Yes
   - No
10. Did you ever suffer an anterior cruciate ligament tear?
    - Yes
    - No

Injury prevention

1. Did you implement exercises to prevent ankle or knee injuries during the preseason (July-August) / in-season (September-November)?
   - Yes
   - No (go to question 20)
2. Did you implement exercises to improve your stability? (see video for an example exercise)
   - Yes
   - No (go to question 15)
3. How often did you implement stability exercises?
   - 1 x/week
   - 2 x/week
   - 3 x/week
   - 4 x/week
   - 5 x/week
   - 1 x/month
   - 2 x/month
   - 3 x/month
4. How much time did you spend on stability exercises during training each time?
   - < 10 minutes
   - > 10 minutes
5. Did you implement exercises to improve your leg strength by means of jumping exercises, also called plyometric exercises? (see video for an example exercise)
   - Yes
   - No
6. How often did you implement plyometric exercises?
   - 1 x/week
   - 2 x/week
   - 3 x/week
   - 4 x/week
   - 5 x/week
   - 1 x/month
   - 2 x/month
   - 3 x/month
7. How much time did you spend on plyometric exercises during training each time?
   - < 10 minutes
   - > 10 minutes
8. If you performed other exercises to prevent ankle and/or knee injuries than described in the questions above, please describe the exercises you performed.
9. Where did you get knowledge about exercises to prevent ankle or knee injuries?
   - Coach
   - Physiotherapist
   - Medical doctor
   - Internet
   - (scientific) journals
   - Info session of the football federation
   - Info brochure of the football federation
   - Other (specify)
   - None of the above
10. Please tick all reasons that apply to you for not performing exercises to prevent ankle or knee injuries.
    - I do not need preventive exercises
    - I do not believe preventive exercises could prevent injuries
    - I have no knowledge of preventive exercises
    - I do not know how to teach preventive exercises
    - I do not have positive experiences from performing preventive exercises in the past
    - A doctor or physiotherapist told me these exercises cannot prevent injuries
    - I have no time for preventive exercises
    - I think that stretching and warming-up are sufficient to prevent injuries
    - Other (specify)

**Appendix 2**. Flow of study participants.


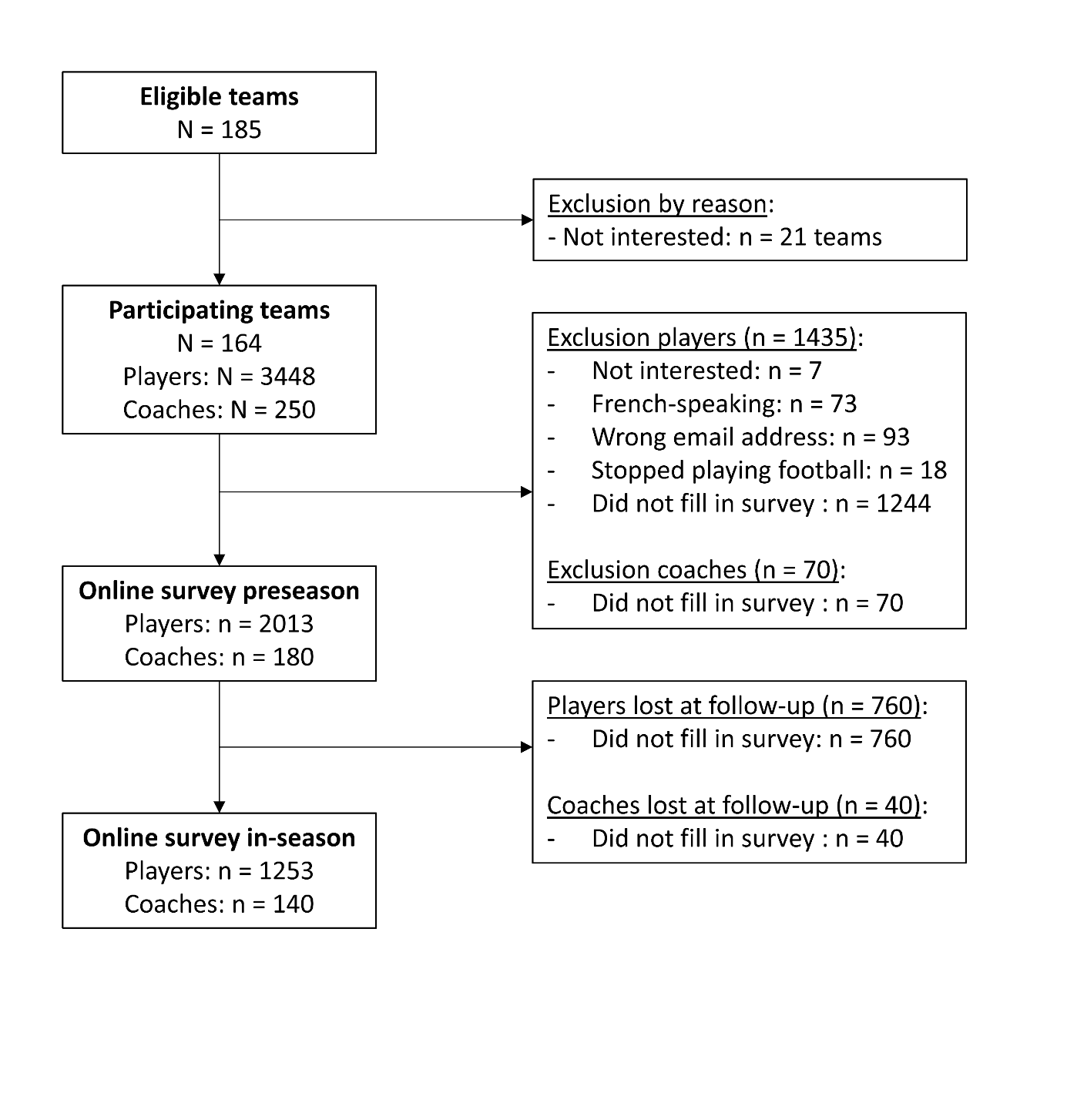

Supplement: Supplementary file 1 — Supplementary file1 (DOCX 1449 KB) [file 167_2022_6878_MOESM1_ESM.docx]
